# Genetic Association Between Angiotensinogen Polymorphisms and Lung Cancer Risk

**DOI:** 10.1097/MD.0000000000001250

**Published:** 2015-09-18

**Authors:** Hong Wang, Kun Zhang, Haifeng Qin, Lin Yang, Liyu Zhang, Yanyan Cao

**Affiliations:** From the Department of Lung Cancer, 307 Hospital of PLA, Affiliated Hospital of Academy of Military Medical Sciences, FengTai Area, Beijing, China.

## Abstract

Earlier published studies investigating the association between polymorphisms in the angiotensinogen gene and lung cancer risk showed no consistent results. In this study, we have summarized all currently available data to examine the correlation by meta-analysis.

Case–control studies addressing the association being examined were identified through Embase, the Cochrane Library, ISI Web of Science (Web of Knowledge), Google Scholar, PubMed, and CNKI databases. Risk of lung cancer (odds ratio [OR] and 95% confidence interval [CI]) was estimated with the fixed or the random effects model assuming homozygous, allele, heterozygous, dominant, and recessive models for all angiotensinogen polymorphisms.

We identified a total of 10 articles in this meta-analysis, including 7 for Leu84Phe, 4 for Ile143Val, and 3 for Leu53Leu. In the meta-analysis of Leu84Phe polymorphism, the homozygous model provided an OR of 1.44 (Phe/Phe vs Ile/Ile: OR = 1.44, 95% CI = 1.04–1.99, *P* values for heterogeneity test (Q-test) [*P*_Het_] = 0.382). The significantly increased risk was similarly indicated in the recessive model (Phe/Phe vs Phe/Ile + Ile/Ile: OR = 1.41, 95% CI = 1.02–1.95, *P*_Het_ = 0.381). We also observed a positive association in the Caucasian subgroup. The heterozygous model and the dominant model tested for the Ile143Val polymorphism showed a marginally increased risk (Ile/Val vs Ile/Ile: OR = 1.16, 95% CI = 1.00–1.36, *P*_Het_ = 0.323; Val/Val + Ile/Val vs Ile/Ile: OR = 1.15, 95% CI = 0.99–1.34, *P*_Het_ = 0.253).

These data suggest that Leu84Phe and Ile143Val polymorphisms in the angiotensinogen gene may be useful biomarkers for lung cancer in some specific populations.

## INTRODUCTION

Lung cancer represents the commonest malignant tumor in humans. An estimation in 2008 showed 1.6 million cases and 1.4 million deaths assigned to this cancer.^1^ People across the global are predicted to be at constant risk of developing lung cancer as a result of the frequent exposure to exogenous and endogenous carcinogens.^2^ Lung cancer is a predominantly smoking-induced cancer.^3^ The appearance of the evidence that approximately 80% of smokers do not develop lung cancer illustrates a significant role of host genetic factors. It has been presumed that identification of these genes, particularly those involved in the repair of deoxyribonucleic acid (DNA) damage caused by environmental hazards, such as tobacco smoke, may help to reduce the incidence and mortality rate.^4^

Dysfunction of the genome maintenance system leads to occurrence of cancer. DNA repair system is a DNA-caretaking mechanism essential for the maintenance of genome stability through repairing DNA damage and thereby prevents oncogenesis.^5^ Tobacco smoke as a dominant component that contributes to the onset of lung cancer contains a long list of carcinogens, and it is the metabolites of these carcinogens that cause the generation of mutagenic O6-alkylguanine DNA adducts,^6^ which play a key role in carcinogenesis, mutagenesis, and apoptotic activities. Angiotensinogen removes the smoking-induced bulky DNA adducts with the aid of an alkyltransferase protein that translocates the methyl group to a specific cysteine acceptor site on the surface of the protein itself.^7^ Animal studies have reported that mice deficient in angiotensinogen are more susceptible to cancer, and elevated cytotoxicity has been detected in the mice exposed to alkylating agents.^8–10^ It has also been reported that the transgenic mice with higher levels of angiotensinogen are at lower risk of developing cancer.^11^ In addition, either high or low levels of angiotensinogen have been observed in various human tumor tissues.^12,13^ These data indicate that the functioning of angiotensinogen is markedly related to the initiation and progression of cancer.

The human angiotensinogen gene on chromosome 10q26 (corresponds to O^6^-methylguanine-DNA methyltransferase, ATG, and *MGMT*), consists of 5 exons and spans 300 kb in size. The polymorphic variability in the genes that participate in the restoration of DNA damage and carcinogen metabolism has been suggested to modulate the transcriptional activity of these genes, predisposing the individuals to varying degrees of cancer.^4,14^ Several polymorphisms, including Leu84Phe, Ile143Val, Leu53Leu, Lys178Arg, and 485C>A, have been implicated in lung cancer, but the most studied has been Leu84Phe, Ile143Val, and Leu53Leu. Studies of genetic susceptibility to lung cancer and the polymorphisms of interest showed inconsistent results.^15–18^ Most importantly, no previous meta-analysis has examined the correlation between the angiotensinogen polymorphisms and lung cancer susceptibility. In view of this, we carried out a quantitative assessment in this study.

## MATERIALS AND METHODS

### Ethics Statement

This study was approved by the PLA 307th Hospital ethics committee. This study not involves patients, ethical approval was not required.

### Inclusion Criteria

The human studies were considered eligible only if they satisfied the criteria listed as follows: an independent case–control or cohort study that examined the association of angiotensinogen polymorphism with risk of lung cancer, and clearly reported the data on frequency of genotypes of the polymorphisms being investigated. When the same case group was investigated in more than 1 publication, we selected the publication with the largest number of participants.

### Search Strategy

To identify the studies satisfying the aforementioned criteria, we carried out a comprehensive literature search in the Embase, the Cochrane Library, ISI Web of Science (Web of Knowledge), Google Scholar, PubMed, and CNKI databases up to January 2014, using the following terms and their synonyms: “angiotensinogen,” “O6-alkylguanine-DNA alkyltransferase,” “O^6^-methylguanine-DNA methyltransferase,” “*MGMT*,” “polymorphism,” “variants,” “genotypes,” and “lung cancer.” As computer-based searches may miss some eligible studies, we manually reviewed the references quoted in the original articles.

### Data Extraction

For the sake of data accuracy, data extraction was performed by 2 investigators independently. The extracted items, including authors, year of publication, polymorphism investigated, country of origin, ethnicity, minor allele frequency in controls when possible, source of controls and DNA sample, genotyping methods, and genotype frequencies between cases and controls, were carefully checked by the 3rd investigator. For the studies that had reported 2 or more ethnic groups, they are treated as an independent population.

### Statistical Analysis

Lung cancer risk was estimated using an odds ratio (OR) and its corresponding 95% confidence interval (CI). The pooled ORs were calculated for homozygous model, allele model, heterozygous model, dominant model, and recessive model for all angiotensinogen polymorphisms. The Z-test was used to evaluate the significance of the summary ORs, with a *P* value less than 0.05 being considered significant.

Statistical heterogeneity was measured using a Chi-square-based Q test (*P* < 0.05 represented significant heterogeneity). If there was no indication of substantial heterogeneity, we chose to use the fixed effects model for the calculation of summary ORs;^19^ otherwise, the random effects model was selected to derive a wider CIs.^20^ Hardy–Weinberg equilibrium (HWE) was checked for all studies in the control groups.^21^ To examine the stability and reliability of the combined estimations, we performed sensitivity analysis by excluding the studies with HWE deviation. In addition, the funnel plots^22^ and Egger linear regression test^23^ were utilized to assess publication bias.

STATA software (version 12.0; Stata Corporation, College Station, TX) was performed to deal with all statistical analyses. All *P* values <0.05 were deemed as significant unless specially stated.

## RESULTS

### Characteristics of Studies

**FIGURE 1:**
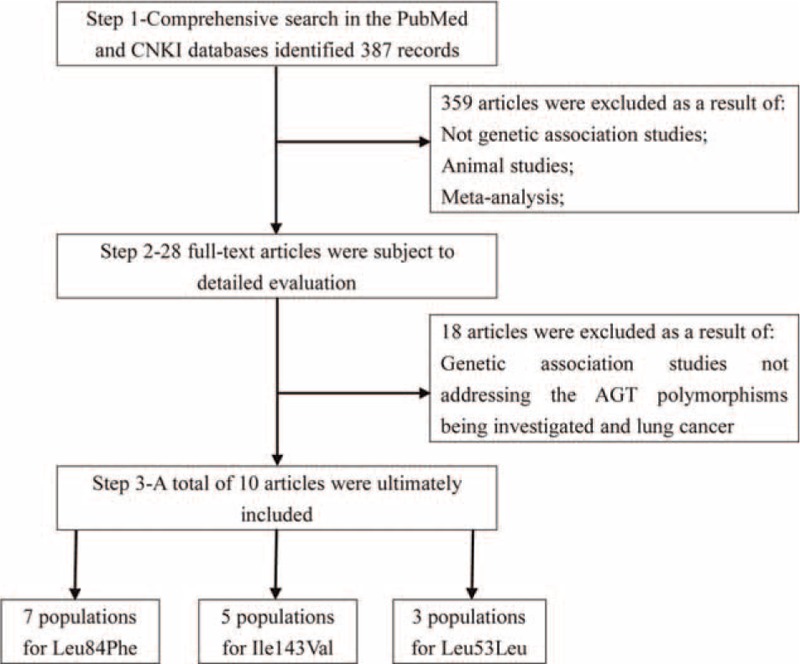
Flow chart showing the detailed selection of studies.

A total of 10 articles^15–18,24–29^ were ultimately included after excluding the nongenetic association studies, and the genetic association studies not addressing the angiotensinogen polymorphisms and lung cancer. A flow diagram of study exclusion and inclusion with specific reasons is shown in Figure 1. The 10 articles provided 7 populations for Leu84Phe, 5 for Ile143Val, and 3 for Leu53Leu. Among the studies of Leu84Phe and Leu53Leu, Caucasians and Asians were employed, while for Ile143Val, African and Caucasian subjects were included. DNA sample was extracted from blood, lung cancer tissue, lymphocytes, or buccal cells. Genotyping for the angiotensinogen polymorphisms was performed using multiple assays, such as single-strand conformation polymorphism, restriction fragment length polymorphism, Taqman, and direct sequencing (Table 1). All studies were in HWE with the exception of Krzesniak et al (*P* = 0.009).

**TABLE 1:**
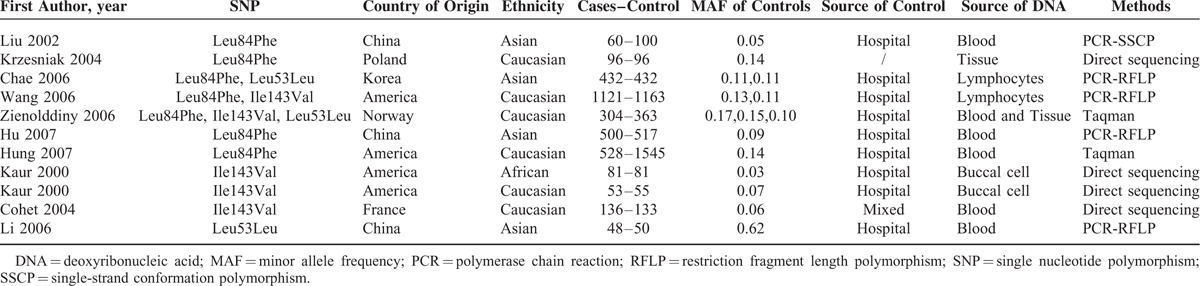
Main Characteristics Summarized for the Studies Included in This Meta-Analysis

### Quantitative Synthesis

The meta-analysis results are shown in Table 2. For Leu84Phe and Ile143Val, the ORs were summarized using the fixed effects model. For Leu53Leu, the values under the homozygous model and the recessive model were calculated by the random effects model, as significant heterogeneity was indicated.

**TABLE 2:**
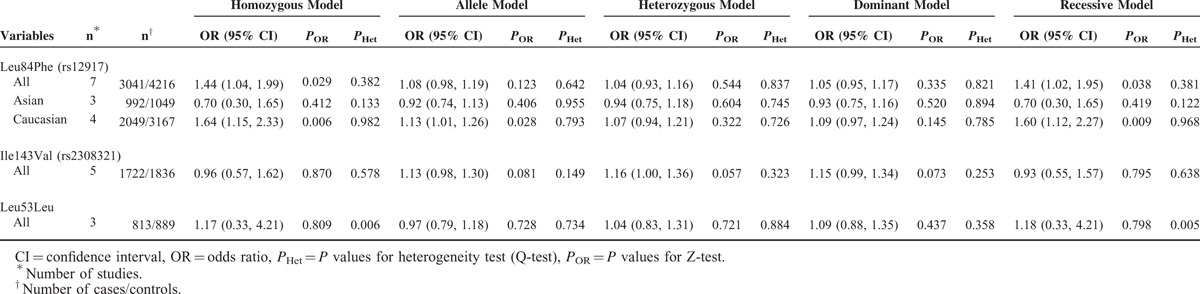
Meta-analysis results for the angiotensinogen polymorphisms and lung cancer risk

### Leu84Phe and Risk of Lung Cancer

**FIGURE 2:**
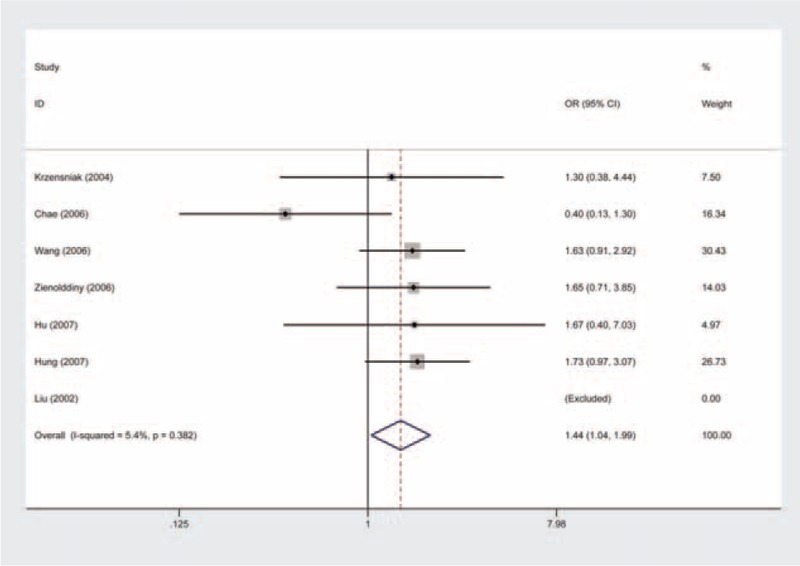
Forest plot for the relation between angiotensinogen gene Leu84Phe polymorphism and risk of lung cancer using the homozygous model (Fixed effects model). The squares and horizontal lines correspond to the study-specific OR and 95% CI. The area of the squares reflects the weight. The diamond represents the summary OR and 95% CI. CI = confidence interval, OR = odds ratio.

**FIGURE 3:**
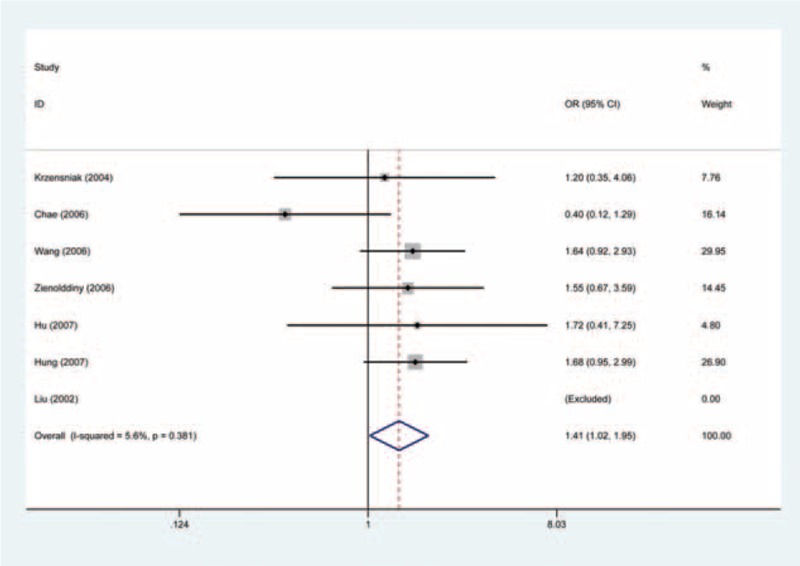
Forest plot for the relation between angiotensinogen gene Leu84Phe polymorphism and risk of lung cancer using the recessive model (Fixed effects model). The squares and horizontal lines correspond to the study-specific OR and 95% CI. The area of the squares reflects the weight. The diamond represents the summary OR and 95% CI. CI = confidence interval, OR = odds ratio.

We carried out both overall analysis and stratified analysis by ethnicity for the Leu84Phe. On the whole, the homozygous model provided an OR of 1.44 (Phe/Phe vs Ile/Ile: OR = 1.44, 95% CI = 1.04–1.99, *P* values for heterogeneity test (Q-test) [*P*_Het_] = 0.382) (Figure 2, Table 2), suggesting the Phe/Phe genotype carriers were more susceptible to lung cancer, as compared with the Ile/Ile genotype carriers. The significantly increased risk was also indicated in the recessive model (Phe/Phe vs Phe/Ile + Ile/Ile: OR = 1.41, 95% CI = 1.02–1.95, *P*_Het_ = 0.381) (Figure 3, Table 2).

When data were restricted to Caucasians, we observed a positive association, which was more pronounced among the Phe/Phe carriers (Phe/Phe vs Ile/Ile: OR = 1.64, 95% CI = 1.15–2.33, *P*_Het_ = 0.982; Phe/Phe vs Phe/Ile + Ile/Ile: OR = 1.60, 95% CI = 1.12–2.27, *P*_Het_ = 0.968) (Table 2), and less pronounced among the individuals with a single Phe allele (Phe vs Ile: OR = 1.13, 95% CI = 1.01–1.26, *P*_Het_ = 0.793) (Table 2).

### Ile143Val and Risk of Lung Cancer

Among the genetic models tested, although we did not found any significant association between the Ile143Val polymorphism and overall risk of lung cancer, the heterozygous model and the dominant model revealed a marginally increased risk (Ile/Val vs Ile/Ile: OR = 1.16, 95% CI = 1.00–1.36, *P*_Het_ = 0.323; Val/Val + Ile/Val vs Ile/Ile: OR = 1.15, 95% CI = 0.99–1.34, *P*_Het_ = 0.253) (Table 2).

### Leu53Leu and Risk of Lung Cancer

We then meta-analyzed the data derived from studies of the Leu53Leu polymorphism and risk of lung cancer. However, none of the genetic models revealed a significant association (Table 2).

### Sensitivity Analysis

Sensitivity analysis was performed for the Leu84Phe polymorphism, as only 1 study concerning this polymorphism showed significant HWE deviation.^16^ We excluded the Polish study and found no substantial alternations in the pooled ORs (Table 3). This suggested the stability of the risk estimations.

**TABLE 3:**
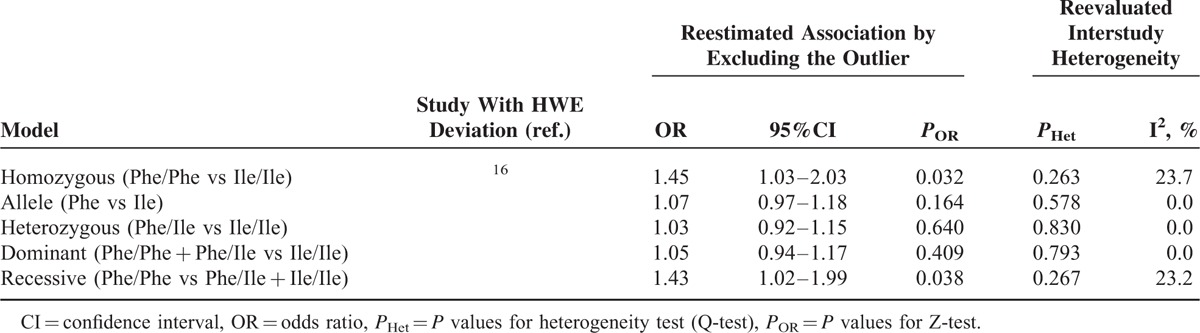
Sensitivity Analysis of Leu84Phe Polymorphism

### Publication Bias

**FIGURE 4:**
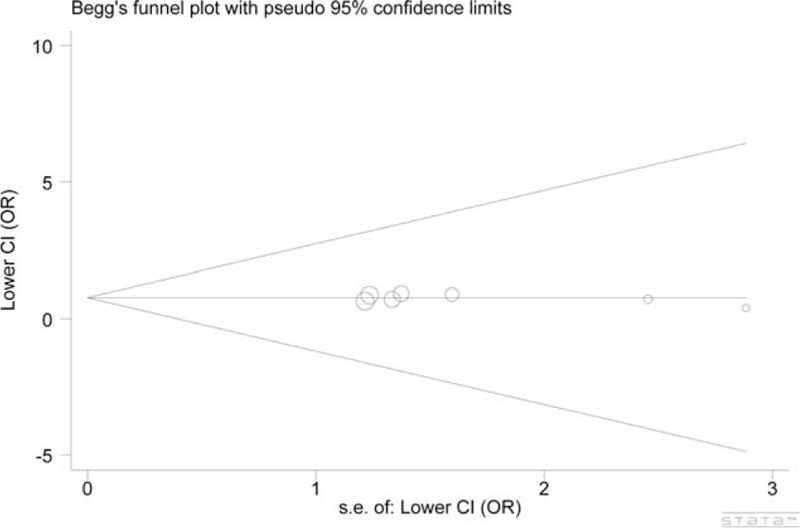
Funnel plot for the dominant model of the Leu84Phe polymorphism indicated that no publication bias existed. Each circle corresponds to 1 study.

**FIGURE 5:**
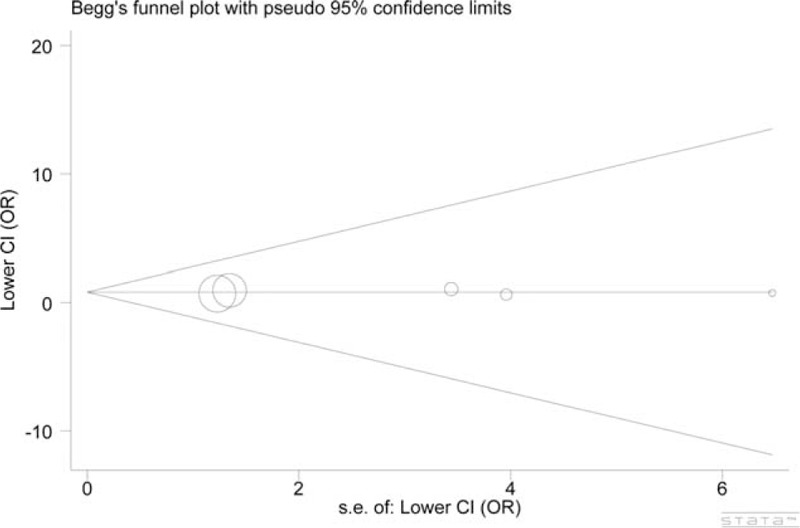
Funnel plot for the allele model of the Ile143Val polymorphism indicated that no publication bias existed. Each circle corresponds to 1 study.

The circles (each corresponds to an independent study) were symmetrically distributed in the funnel plots of Leu84Phe and Ile143Val polymorphisms. The Egger test provided statistical evidence supporting the symmetry of funnel plots, suggesting there was no substantial publication bias in our meta-analysis. Figures 4 and 5 show the funnel plots of the former polymorphism (dominant model: *P* = 0.387) and of the latter polymorphism (allele model: *P* = 0.950), respectively.

## DISCUSSION

The *ATG* gene is of special interest in the past decade for its capability of repairing DNA alkylation adducts induced by tobacco smoke-specific alkylating agents,^30,31^ and the polymorphisms within the region have been reportedly to decrease the activity in the repair of bulky alkylated DNA adducts.^32^ Many groups are promoted to investigate the role of *ATG* polymorphisms in lung carcinogenesis. However, these studies showed no consistent results. For example, the earliest study suggested a slight but significant correlation between *ATG* Leu84Phe polymorphism and risk of lung cancer in a sample of Chinese patients.^15^ Several follow-up studies succeeded in replicating this finding,^18,25^ but many others failed to.^16,17^ Similarly, the inconsistent associations could be seen in the previous studies of Ile143Val polymorphism.^24,27^ Differences in genetic background and the relatively small number of individuals in the published studies are 2 possible aspects that contribute to this divergency. Therefore, the exact role of *ATG* polymorphisms in lung cancer remains to be elucidated.

The limited statistical power caused by sample insufficiency of the independent studies can be enhanced when a meta-analysis is performed. In our meta-analysis, data from 10 case–control studies were summarized to assess the association between *ATG* polymorphisms and lung cancer risk. We found significantly increased susceptibility of lung cancer among Phe/Phe genotype carriers of the Leu84Phe polymorphism. A similar trend was revealed in the subgroup of Caucasian populations, but not in Asian populations. For the Ile143Val polymorphism, we only observed a marginal increase. However, no association was suggested for the Leu53Leu polymorphism. Recently, a meta-analysis has assessed risk of lung cancer in relation to the Leu84Phe polymorphism, and suggested the presence of an association among Caucasians. In this study, Qiu et al^33^ stated that all included studies were in HWE. This statement contradicts the fact that genotype distribution in controls of the Polish study by Krzesniak et al violated the law of HWE (*P* = 0.009). The violation possibly related to the small population size could cause deviations from the expected values. Besides, lung cancer is a polygenic disease, the true association with candidate genes can hardly be determined using a single polymorphism. The present study, with a total of 5260 additional participants, meta-analyzed 3 well-characterized polymorphisms in the *ATG* gene that was suggested to increase the susceptibility of lung cancer.

Previous functional studies have provided a sound theoretical basis for the present finding. It has been found that epigenetic inactivation of ATG by promoter hypermethylation may cause mutations in *K-ras* and *p53*, a critical process that initiates tumorigenesis.^34,35^ Accumulated evidence also indicated a substantial role of *ATG* polymoprhisms in the protein function. Teo et al^37^ and Pegg et al^38^ suggested that alternations at position 84, such as the alternation to phenylalanine, abrogate the ability of the alkylated ATG to bind the estrogen receptor, leading to the failure to prevent tumor growth. More recently, Hill et al^39^ similarly reported that the Leu84Phe polymorphism may affect the phenotype of the ATG protein, and thereby attenuates the capability to repair alkylating agents-associated damage. These data suggest a possible linkage between lung cancer and the *ATG* gene polymoprhisms.

It is intriguing that the results indicated increased susceptibility to lung cancer among Caucasians, rather than Asians. There are 2 major explanations. First, the number of Caucasian participants is obviously different from that of Asian participants (2049 cases/3167 controls vs 992 cases/1049 controls). The detection power may be limited by the limited sample size. Second, frequency of the Phe allele between Caucasians and Asians is not equal (15.1% vs 9.7%). It has been suggested that the odds to develop lung cancer in general population are associated with the presence of low-penetrance, high-frequency polymorphisms.^36^ Lung cancer may particularly favor Caucasians, because of the higher frequency of the Phe allele of Leu84Phe polymorphism.

There are several shortcomings in this meta-analysis. The first shortcoming refers to the inadequate sample size of the polymorphisms being investigated, which may decrease the precision of the estimations and lead to biased results. The second limitation is the overgeneralization of the associations. There is evidence that angiotensinogen polymorphisms play a major role in lung cancer susceptibility among never-smokers,^28^ highlighting the importance to identify other potential susceptible agents for lung cancer in addition to smoking. The third shortcoming is publication bias. The literature search has identified all published papers. But we have no access to the unpublished records. Therefore, publication bias may have been introduced, although it is short of statistical significance in this study.

In conclusion, this meta-analysis provided statistical evidence supporting that the angiotensinogen Leu84Phe polymorphism was associated with increased risk of lung cancer, especially in Caucasian populations. A marginal association was also revealed in the analysis of Ile143Val polymorphism. Future studies with a sufficiently large number of participants are required to substantiate the previously reported associations.

## References

[1] Jemal A Bray F Center MM Global cancer statistics. *CA Cancer J Clin* 2011; 61:69–90. 21296855 10.3322/caac.20107

[2] Xie Y Minna JD Predicting the future for people with lung cancer. *Nat Med* 2008; 14:812–813. 18685594 10.1038/nm0808-812 PMC2833359

[3] IARC. Tobacco Smoke and Involuntary Smoking. IARC Monographs on the Evaluation of Carcinogenic Risks to Humans. 2004; Lyon, France: IARC, 83:1–1438. PMC4781536 15285078

[4] Spitz MR Wei Q Dong Q Genetic susceptibility to lung cancer: the role of DNA damage and repair. *Cancer Epidemiol Biomarkers Prev* 2003; 12:689–698. 12917198

[5] Hoeijmakers JH Genome maintenance mechanisms for preventing cancer. *Nature* 2001; 411:366–374. 11357144 10.1038/35077232

[6] Bartsch H Montesano R Relevance of nitrosamines to human cancer. *Carcinogenesis* 1984; 5:1381–1393. 6386215 10.1093/carcin/5.11.1381

[7] Sedgwick B Repairing DNA-methylation damage. *Nat Rev Mol Cell Biol* 2004; 5:148–157. 15040447 10.1038/nrm1312

[8] Tsuzuki T Sakumi K Shiraishi A Targeted disruption of the DNA repair methyltransferase gene renders mice hypersensitive to alkylating agent. *Carcinogenesis* 1996; 17:1215–1220. 8681434 10.1093/carcin/17.6.1215

[9] Sakumi K Shiraishi A Shimizu S Methylnitrosourea-induced tumorigenesis in MGMT gene knockout mice. *Cancer Res* 1997; 57:2415–2418. 9192819

[10] Pegg AE Repair of O(6)-alkylguanine by alkyltransferases. *Mutat Res* 2000; 462:83–100. 10767620 10.1016/s1383-5742(00)00017-x

[11] Zhou ZQ Manguino D Kewitt K Spontaneous hepatocellular carcinoma is reduced in transgenic mice overexpressing human O6- methylguanine-DNA methyltransferase. *Proc Natl Acad Sci U S A* 2001; 98:12566–12571. 11606727 10.1073/pnas.221232998 PMC60094

[12] Wani G Wani AA D’Ambrosio SM In situ hybridization of human kidney tissue reveals cell-type-specific expression of the O6-methylguanine-DNA methyltransferase gene. *Carcinogenesis* 1992; 13:463–468. 1547538 10.1093/carcin/13.3.463

[13] Citron M Decker R Chen S O6-methylguanine-DNA methyltransferase in human normal and tumor tissue from brain, lung, and ovary. *Cancer Res* 1991; 51:4131–4134. 1868433

[14] Hecht SS Cigarette smoking and lung cancer: chemical mechanisms and approaches to prevention. *Lancet Oncol* 2002; 3:461–469. 12147432 10.1016/s1470-2045(02)00815-x

[15] Liu RQ Zhang ZX Single-nucleotide polymorphisms of human O6-methylguanine-DNA methyltransferase (MGMT) gene in lung cancer patients from south China. *Wei Sheng Du Li Xue Za Zhi* 2002; 16:1–5.

[16] Krzesniak M Butkiewicz D Samojedny A Polymorphisms in TDG and MGMT genes - epidemiological and functional study in lung cancer patients from Poland. *Ann Hum Genet* 2004; 68 (Pt 4):300–312. 15225156 10.1046/j.1529-8817.2004.00079.x

[17] Chae MH Jang JS Kang HG O6-alkylguanine-DNA alkyltransferase gene polymorphisms and the risk of primary lung cancer. *Mol Carcinog* 2006; 45:239–249. 16385589 10.1002/mc.20171

[18] Wang L Liu H Zhang Z Association of genetic variants of O6-methylguanine-DNA methyltransferase with risk of lung cancer in non-Hispanic Whites. *Cancer Epidemiol Biomarkers Prev* 2006; 15:2364–2369. 17164358 10.1158/1055-9965.EPI-06-0437

[19] Mantel N Haenszel W Statistical aspects of the analysis of data from retrospective studies of disease. *J Natl Cancer Inst* 1959; 22:719–748. 13655060

[20] Venkateshan SP Sidhu S Malhotra S Efficacy of biologicals in the treatment of rheumatoid arthritis: a meta-analysis. *Pharmacology* 2009; 83:1–9. 18957873 10.1159/000165777

[21] Hernandez JL Weir BS A disequilibrium coefficient approach to Hardy-Weinberg testing. *Biometrics* 1989; 45:53–70. 2720060

[22] Begg CB Mazumdar M Operating characteristics of a rank correlation test for publication bias. *Biometrics* 1994; 50:1088–1101. 7786990

[23] Egger M Davey Smith G Schneider M Bias in meta-analysis detected by a simple, graphical test. *BMJ* 1997; 315:629–634. 9310563 10.1136/bmj.315.7109.629 PMC2127453

[24] Zienolddiny S Campa D Lind H Polymorphisms of DNA repair genes and risk of non-small cell lung cancer. *Carcinogenesis* 2006; 27:560–567. 16195237 10.1093/carcin/bgi232

[25] Hu Z Wang H Shao M Genetic variants in MGMT and risk of lung cancer in Southeastern Chinese: a haplotype-based analysis. *Hum Mutat* 2007; 28:431–440. 17285603 10.1002/humu.20462

[26] Hung RJ Baragatti M Thomas D Inherited predisposition of lung cancer: a hierarchical modeling approach to DNA repair and cell cycle control pathways. *Cancer Epidemiol Biomarkers Prev* 2007; 16:2736–2744. 18086781 10.1158/1055-9965.EPI-07-0494

[27] Kaur TB Travaline JM Gaughan JP Role of polymorphisms in codons 143 and 160 of the O6-alkylguanine DNA alkyltransferase gene in lung cancer risk. *Cancer Epidemiol Biomarkers Prev* 2000; 9:339–342. 10750675

[28] Cohet C Borel S Nyberg F Exon 5 polymorphisms in the O6-alkylguanine DNA alkyltransferase gene and lung cancer risk in non-smokers exposed to second-hand smoke. *Cancer Epidemiol Biomarkers Prev* 2004; 13:320–323. 14973087 10.1158/1055-9965.epi-03-0120

[29] Li J Shao G Liu L Association of polymorphism of codon 72 in p53 gene with susceptibility and radiosensitivity of non-small cell lung cancer in Chinese population. *Chinese J Lung Cancer* 2006; 9:173–176. 10.3779/j.issn.1009-3419.2006.02.15 21144305

[30] Montesano R Becker R Hall J Repair of DNA alkylation adducts in mammalian cells. *Biochimie* 1985; 67:919–928. 3910113 10.1016/s0300-9084(85)80288-1

[31] Edara S Kanugula S Pegg AE Expression of the inactive C145A mutant human O6-alkylguanine-DNA alkyltransferase in E.coli increases cell killing and mutations by N-methyl-N′-nitro-N-nitrosoguanidine. *Carcinogenesis* 1999; 20:103–108. 9934856 10.1093/carcin/20.1.103

[32] Edara S Kanugula S Goodtzova K Resistance of the human O6-alkylguanine-DNA alkyltransferase containing arginine at codon 160 to inactivation by O6-benzylguanine. *Cancer Res* 1996; 56:5571–5575. 8971155

[33] Qiu ZX Xue F Shi XF MGMT Leu84Phe gene polymorphism and lung cancer risk: a meta-analysis. *Tumour Biol* 2014; 35:4381–4387. 24390665 10.1007/s13277-013-1576-3

[34] Esteller M Toyota M Sanchez-Cespedes M Inactivation of the DNA repair gene O6-methylguanine-DNA methyltransferase by promoter hypermethylation is associated with G to A mutations in K-ras in colorectal tumorigenesis. *Cancer Res* 2000; 60:2368–2371. 10811111

[35] Esteller M Risques RA Toyota M Promoter hypermethylation of the DNA repair gene O(6)-methylguanine-DNA methyltransferase is associated with the presence of G:C to A:T transition mutations in p53 in human colorectal tumorigenesis. *Cancer Res* 2001; 61:4689–4692. 11406538

[36] Kiyohara C Yoshimasu K Takayama K Lung cancer susceptibility: are we on our way to identifying a high-risk group? *Future Oncol* 2007; 3:617–627. 18041914 10.2217/14796694.3.6.617

[37] Teo AK Oh HK Ali RB The modified human DNA repair enzyme O6-methylguanine-DNA methyltransferase is a negative regulator of estrogen receptor-mediated transcription upon alkylation DNA damage. *Mol Cell Biol* 2001; 21:7105–7114. 11564893 10.1128/MCB.21.20.7105-7114.2001 PMC99886

[38] Pegg AE Fang Q Loktionova NA Human variants of O6-alkylguanine-DNA alkyltransferase. *DNA Repair (Amst)* 2007; 6:1071–1078. 17482892 10.1016/j.dnarep.2007.03.012 PMC2030992

[39] Hill CE Wickliffe JK Guerin AT The L84F polymorphism in the O6-Methylguanine-DNA-Methyltransferase (MGMT) gene is associated with increased hypoxanthine phosphoribosyltransferase (HPRT) mutant frequency in lymphocytes of tobacco smokers. *Pharmacogenet Genomics* 2007; 17:743–753. 17700363 10.1097/FPC.0b013e3281111eb1

